# Protocol for decoding immune predictors of response to immunotherapy through pan-cancer multiomics analysis

**DOI:** 10.1016/j.xpro.2025.104183

**Published:** 2025-11-03

**Authors:** Xinyue Jian, Xu Zheng, Kun Jiao, Weiyuan Li, Xuexin Li

**Affiliations:** 1 Department of General Surgery, The Fourth Affiliated Hospital, China Medical University, Shenyang, Liaoning 110032, China; 2 Key Laboratory of Precision Diagnosis and Treatment of Gastrointestinal Tumors, Ministry of Education, China Medical University, Shenyang, Liaoning 110122, China; 3 Institute of Health Sciences, China Medical University, Shenyang, Liaoning 110122, China; 4 Department of Physiology and Pharmacology, Karolinska Institutet, 171 65 Solna, Sweden; 5 Hospital of Yunnan Province, Kunming, Yunnan Province 650000, China; 6 The Affiliated Hospital of Kunming University of Science and Technology, Kunming, Yunnan Province 650000, China

**Keywords:** Bioinformatics, Cell Biology, Mass Cytometry, Cancer, RNA-seq, Immunology

## Abstract

Here, we present a protocol for analyzing T cell dynamics in multiple cancers through single-cell RNA sequencing (scRNA-seq), single-cell immune profiling, and mass cytometry/cytometry by time-of-flight (CyTOF). We describe steps for performing gene-regulatory network analysis to identify key transcription factors and cell-cell interaction analysis to explore immune signaling. This protocol facilitates the identification of T cell subsets associated with immune checkpoint blockade (ICB) response and the development of predictive biomarkers.

For complete details on the use and execution of this protocol, please refer to Li et al.^1^

## Highlights

• Steps for integrating multi-omics data to analyze T cell dynamics across cancers
• Instructions for single-cell RNA sequencing from sample collection to data analysis
• Guidance on TCR clonality analysis to track T cell response to ICB therapy
• Steps for building a response prediction model and response index (RI) for ICB therapy

## Abstract (editor highlights)

Publisher’s note: Undertaking any experimental protocol requires adherence to local institutional guidelines for laboratory safety and ethics.

## Graphical abstract

**Figure:**
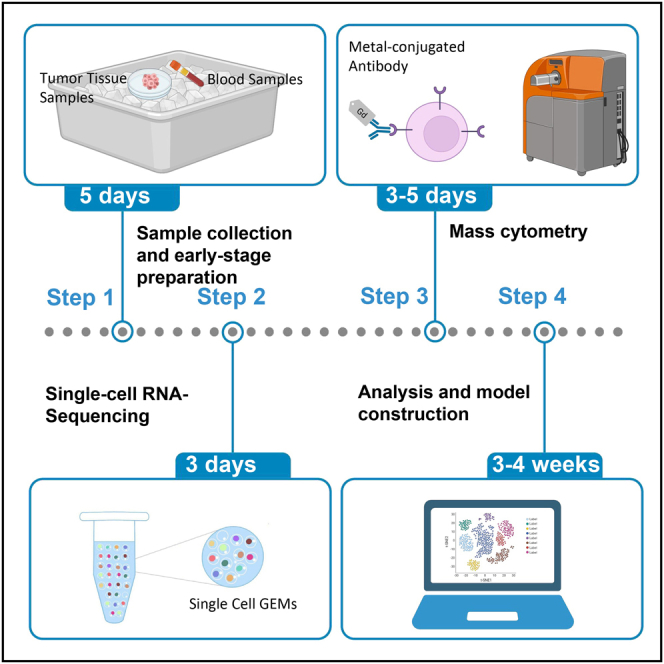

## Teaser

Here, we present a protocol for analyzing T cell dynamics in multiple cancers through single-cell RNA sequencing (scRNA-seq), single-cell immune profiling, and mass cytometry (CyTOF). We describe steps for performing gene-regulatory network analysis to identify key transcription factors and cell-cell interaction analysis to explore immune signaling. This protocol facilitates the identification of T cell subsets associated with immune checkpoint blockade (ICB) response and the development of predictive biomarkers.

## Before you begin

The primary scientific objective of this protocol is to characterize the factors influencing immune checkpoint blockade (ICB) response across multiple cancer types. This protocol will identify T cell subsets that correlate with ICB response and resistance by analyzing T cell dynamics using single-cell RNA sequencing and mass cytometry (CyTOF). We will perform gene regulatory network analysis to identify clinically relevant transcription factors and their associated regulatory networks. Cell-cell interaction analysis will systematically map interactions between T cells and neighboring immune cells to explore immune signaling. T cell receptor (TCR) repertoire analysis will examine clonal expansion patterns associated with ICB response. Finally, we will integrate these data to construct a response prediction model that can identify patients likely to respond to ICB therapy. Additionally, this protocol can be applied to identify new biomarkers for other diseases, guide the development of new immunotherapies or combination therapies, design and monitor clinical trials, and address fundamental research questions in immunology and cancer biology. The comprehensive single-cell multiomics approach provides a versatile framework for uncovering new biological mechanisms and pathways, enhancing its utility and impact across various research and clinical applications.^2^

### Innovation

This protocol establishes an innovative pan-cancer multiomics framework that systematically integrates single-cell transcriptomics, high-dimensional proteomics via mass cytometry, and T-cell receptor profiling to decipher immune predictors of immunotherapy response. Unlike conventional approaches focusing on single modalities or cancer types, our method synergistically combines transcriptional, protein expression, and clonal dynamics within a unified analytical workflow. A key advancement is our customized computational pipeline that enables integrated analysis of these disparate data types, identifying coordinated changes across molecular layers—including gene regulatory networks, cell-cell communication, and TCR clonal expansion patterns specifically associated with treatment outcomes. Furthermore, we introduce a machine learning-derived Response Index that quantitatively integrates these multi-omics features into a single predictive metric, providing a clinically translatable tool for patient stratification. The protocol is optimized for practical implementation with detailed troubleshooting and quality control procedures for challenging clinical samples, ensuring robustness and reproducibility. This integrated framework represents a substantial advancement over conventional single-modality analyses, offering a more comprehensive approach for biomarker discovery and mechanistic understanding of immunotherapy response and resistance.

### Institutional permissions

The protocol received ethical clearance from Yunnan Cancer Center’s Institutional Review Board and complied strictly with the Declaration of Helsinki’s ethical standards.

Prior to conducting any experiments, all necessary ethical approvals and informed consents were obtained in compliance with institutional and international guidelines.

### Pre-sampling preparation

**Timing: 2 days**

***Note:*** Patient Recruitment and Inclusion Criteria.

1. General Principles for Recruitment. Patients should be selected based on the specific research objectives, with consideration given to:
  a. Histopathological confirmation of certain cancer.
  b. Completion of therapy as defined by the study protocol.
  c. Clinical suitability for biopsy procedures.
  d. Adequate organ function and life expectancy to complete the study.
2. Key Considerations for Exclusion Criteria. To minimize confounding effects, studies should explicitly exclude:
  a. Patients receiving concurrent therapies that may interact with your research objectives (including but not limited to chemotherapy, targeted therapies, or other immunomodulators).
  b. Those participating in other interventional clinical trials during the study period.
3. Additional exclusion criteria should be study-specific and justified based on:
  a. Potential pharmacokinetic interactions.
  b. Overlapping toxicities.
  c. Compromised treatment response assessment.

### Preliminary data analysis preparation

**Timing: 1 day**

4. Hardware Requirements:
  a. Ensure access to a high-performance computing cluster or a powerful workstation capable of handling large-scale single-cell sequencing data.
  b. Prepare sufficient storage space for storing raw and processed data.
5. Software and R Package Installation:
  a. Install R (version 4.2.1 or later) and R Studio.
  b. Install the following R packages for data analysis:
    i. Seurat (version 4.2.1) for single-cell RNA-seq data integration, clustering, dimensionality reduction, and visualization.
    ii. ComplexHeatmap for highly customizable heatmap generation with support for complex annotations and multi-omics data integration.^3^
    iii. AUCell for computes gene set enrichment scores (Area Under the Curve) to evaluate pathway activity or cellular states in single-cell data.
    iv. scFunctions to provide utility functions for single-cell data processing, analysis, and visualization.
    v. rcartocolor to offers Carto color palettes, designed for clear and effective data visualization, particularly in spatial and scientific contexts.iii.Monocle2 for pseudotime trajectory analysis.
    vi. Liana for cell-cell communication analysis.^4^
    vii. Install other necessary R packages for data visualization and statistical analysis.
6. Data Storage and Management:
  a. Set up a secure and organized system for storing raw and processed data.
  b. Regularly back up all data to prevent data loss.
  c. Use version control systems (e.g., Git) to manage and track changes in analysis scripts.

## Key resources table

**Table:** 

REAGENT or RESOURCE	SOURCE	IDENTIFIER
**Antibodies**		

Fc receptor blocking solution (human)	BioLegend	Cat#422302
Anti-CD45 antibody (1 μg/10^6^ cell)	BioLegend	Cat#304002
eBioscience Foxp3/transcription factor staining buffer set	Thermo Fisher Scientific	Cat#00-5523-00
Maxpar antibody labeling kit	Fluidigm	Cat#201300

**Biological samples**		

Peripheral blood sample of CRC patients	This paper	N/A
Human normal colon tissue	This paper	N/A
Human CRC tumor tissue	This paper	N/A

**Chemicals, peptides, and recombinant proteins**		

sCelLiVE preservation solution	Singleron	Cat#1010012
Fluorescent cell analysis dyes (AO/PI)	Sigma-Aldrich	Cat#Z764787
1X PBS buffer	N/A	N/A
1X HBSS buffer	N/A	N/A
BSA	Sigma-Aldrich	Cat#V900933
DAPI (1 μg/mL)	BioLegend	Cat#422801
RBC lysis buffer	Roche	Cat#11814389001
ACK lysing buffer	Thermo Fisher Scientific	Cat#A1049201
FBS	Sigma-Aldrich	Cat#F0193
DMSO	Sigma-Aldrich	Cat#34869
Trypan blue solution	Solarbio	Cat#C0040
Maxpar Fix and Perm buffer	Fluidigm	Cat#201067
PMA	Sigma-Aldrich	Cat#P8139
Ionomycin	Sigma-Aldrich	Cat#10634
Tuning solution	Fluidigm	Cat#201072
EQ beads	Standard BioTools (formerly Fluidigm)	Cat#201078
Bond-Breaker TCEP	Thermo Fisher Scientific	Cat#77720
BioStab antibody stabilizer	Sigma-Aldrich	Cat#55514
Ficoll-Paque PLUS	GE Healthcare	Cat#17-1440-03
RPMI-1640 medium	BasalMedia	Cat#L220KJ
Cell stimulation cocktail (500X)	Invitrogen	Cat#00-4970-93
Protein transport inhibitor cocktail (500X)	Invitrogen	Cat#00-4980-93
Cell stimulation cocktail (plus protein transport inhibitors) (500X)	Invitrogen	Cat#00-4975-93
Phosphate-buffered saline	GENOM	Cat#GNM20012
Bovine serum albumin	Sigma-Aldrich	Cat#V900933
Cell-ID Intercalator-Ir (191/193)	Fluidigm	Cat#201192B
Cell-ID Cisplatin-194Pt	Fluidigm	Cat#201194

**Critical commercial assays**		

Single-cell V(D)J enrichment kit, human T cell	10× Genomics	PN-1000005
Single-cell 5′ library and gel bead kit	10× Genomics	PN-1000006
Single-cell V(D)J enrichment kit, human B cell	10× Genomics	PN-1000016
eBioscience Foxp3/transcription factor staining buffer set	Thermo Fisher Scientific	Cat#00-5523-00

**Deposited data**		

Sequencing data of breast cancer patients	EGA	EGAD00001006608
Sequencing data of BCC patients	GEO	GEO: GSE123813
Sequencing data of ccRCC patients	SRA	SRZ190804
Sequencing data of iCCA patients	GEO	GEO: GSE151530
Sequencing data of HNSCC patients	GEO	GEO: GSE200996
Sequencing data of HCC patients	GEO	GEO: GSE151530
Sequencing data of MIBC patients	GEO	GEO: GSE149652
Sequencing data of SCC patients	GEO	GEO: GSE123813
Sequencing data of NSCLC patients	GEO	GEO: GSE176021
Sequencing data of normal colon	GEO	GEO: GSE144469
Sequencing data of normal bladder	GEO	GEO: GSE159929
Sequencing data of normal skin	GEO	GEO: GSE176021
Sequencing data of normal kidney	GEO	GEO: GSE139555
Sequencing data of normal lung	GEO	GEO: GSE139555
Raw and processed sequencing data of CRC patients	This paper	PRJCA016919
CyTOF data of CRC patients	This paper	PRJCA016919

**Software and algorithms**		

Microsoft Excel	Microsoft	http://products.office.com/en-us/excel
R Studio	The R Project for Statistical Computing	http://www.r-project.org/
Seurat	v.4.2.1	http://satijalab.org/seurat/articles/install.html
Cellranger	10× Genomics v.6.1.0	http://github.com/10XGenomics/cellranger

**Other**		

Pasteur pipettes (5 mL)	N/A	N/A
Ophthalmic scissors/forceps	N/A	N/A
1.5 mL low-adhesion centrifuge tubes	Sigma-Aldrich	Cat#HS4323
15 mL low-adhesion centrifuge tubes	Sigma-Aldrich	Cat#CLS430791
50 mL low-adhesion centrifuge tubes	Sigma-Aldrich	Cat#CLS430829
6 cm sterile culture dishes	N/A	N/A
Pasteur pipettes (5 mL)	N/A	N/A
Fluorescence inverted microscope	N/A	N/A
High-speed refrigerated centrifuge	N/A	N/A
Thermostatic shaking incubator	N/A	N/A
Fluorescence-based cell counter	N/A	N/A
Single-channel pipettes (various ranges)	N/A	N/A
Helios mass cytometer	Fluidigm	N/A
Sterile forceps	N/A	N/A
40 μm cell strainer	Falcon	Cat#352340
100 μm cell strainer	Falcon	Cat#352360
Round-bottom polystyrene tubes with 35 μm cell strainer	Falcon	Cat#352235
FACSAria sorter	BD Biosciences	N/A
100 μm nozzle	BD FACSAria	N/A
CountStar	N/A	N/A
Agilent 4200 Bioanalyzer system	N/A	N/A
Centrifugal filter unit, 3 kDa	Millipore	Cat#UFC500396
Centrifugal filter unit, 50 kDa	Millipore	Cat#UFC505008
10× Genomics Chromium platform	10× Genomics	Chromium Controller
Next-generation sequencing system	Illumina	NovaSeq 6000
Thermal cycler	Bio-Rad	C1000 Touch
Agilent Bioanalyzer 4200	Agilent	Cat#G2991A
Qubit fluorometer	Thermo Fisher Scientific	N/A
Centrifuge	Eppendorf	Cat#5810R
10× Genomics magnetic separator	10× Genomics	PN-120250
10× Genomics vortex adapter	10× Genomics	PN-120251
10× Genomics chip holder	10× Genomics	PN-120252
A mass spectrometry flow cytometry	Fluidigm	N/A

## Materials and equipment

**Table:** FACS buffer (Freshly prepared)

Reagent	Final concentration	Amount
BSA/FBS	0.5%	0.5 g
1 × PBS buffer	N/A	100 mL
EDTA	1 mM	1 mL of 0.1 M
NaN_3_	0.05%	0.05 g
**Total**	**N/A**	**N/A**

Store at 4°C (≤1 month, sterile-filtered).

**Table:** Cisplatin solution

Reagent	Final concentration	Amount
Cell-ID Cisplatin-194Pt(1 mM)	250 nM	0.25 μL
1 × PBS buffer	N/A	1000 μL
**Total**	**N/A**	**1000.25 μL**

Cell-ID Cisplatin-194Pt (1 mM) should be stocked at −20°C, light-protected. Prepare fresh before use.

**Table:** Intercalator solution

Reagent	Final concentration	Amount
Cell-ID Intercalator-Ir	250 nM	0.5 μL
Maxpar Fix and Perm Buffer	N/A	1000 μL
**Total**	**N/A**	**1000.5 μL**

Cell-ID Intercalator-Ir should be stocked at −20°C, light-protected. Freshly diluted before use.

**Table:** Fc Receptor Blocking solution

Reagent	Final concentration	Amount
Human IgG	1 μg/100 μL	1 μg
Mouse IgG	1 μg/100 μL	1 μg
Rat IgG	1 μg/100 μL	1 μg
Hamster IgG	1 μg/100 μL	1 μg
1× PBS buffer	N/A	100 μL
**Total**	**N/A**	**N/A**

Stock at −20°C (IgG). Fresh working solution.

**Table:** Foxp3 Fixation/Permeabilization buffer

Reagent	Final concentration	Amount
eBioscience Fix/Perm Concentrate (4×)	1×	1 mL
eBioscience Fix/Perm Diluent	N/A	3 mL
**Total**	**N/A**	**4 mL**

Store at 4°C. Prepare fresh working solution.

## Step-by-step method details

### Sample collection

**Timing: 2 days**

This initial section describes the procedures for obtaining high-quality clinical specimens, which is the foundation for all subsequent molecular and cellular analyses.

***Note:*** Sample collection included buffer time to account for operational variables.

1. Collect peripheral blood samples and tumor tissue samples from patients, covering pre-treatment and post-treatment specimen collections.
2. Ensure that the sample collection process complies with ethical requirements and obtain written informed consent from the patients.
3. Samples should be processed within 72 hours post-collection, with immediate processing (within 1 hour) strongly recommended for optimal results. Process the samples as soon as possible after collection to maintain cell viability.

***Note:*** All collections have to be performed in accordance to the ethically approved protocols. If immediate processing is possible, tissue preservation can be omitted. If immediate processing is not feasible, preservation in SCelLiVe solution ensures tissue integrity and cell viability until processing.

### Tissue sample preservation

**Timing: 1 day**

This section describes the protocol for stabilizing tissue specimens when immediate processing is not feasible.

4. Thaw the tissue preservation solution (SCelLiVe preservation solution) and keep it on ice until fully liquefied (no ice crystals remaining).
5. Inspect the tissue for microbial contamination, necrotic regions, or residual blood.

***Note:*** If such regions are present, carefully trim and remove them using sterile scissors or forceps. If needed, wash the viable tissue fragments with chilled PBS 3 times before proceeding.

6. Mince the tissue into fragments ≤ 0.5 cm in diameter (roughly 100 mg).

***Note:*** Whenever possible, proceed directly to downstream processing. If immediate processing is not feasible, transfer one tissue fragment into a tube containing SCelLiVe preservation solution. Oversized specimens may compromise cell viability.

***Note:*** Fresh tissue should preferably be processed immediately to ensure optimal cell viability. When processing cannot be performed within 1–2 hours, SCelLiVe preservation solution enables storage/transport at 2°C–8°C for up to 72 hours.

### Tissue sample dissociation

**Timing: 1 day**

This section describes the enzymatic and mechanical process of breaking down solid tumor tissue into a single-cell suspension. The goal is to maximize cell yield and viability while preserving cell surface markers for subsequent staining and sequencing.

***Note:*** Pre-dissociation preparation: Prior to tissue dissociation, ensure the SCelLiVe tissue dissociation solution is equilibrated to 25°C, while PBS and HBSS should be pre-chilled on ice. Preheat the thermostatic shaker to 37°C with rotation speed set at 180 rpm. All dissection instruments including ophthalmic scissors and forceps must be sterilized, and cell counting reagents (trypan blue and fluorescent staining solutions) should be prepared in advance.

7. Transfer fresh tissue to a dish containing HBSS buffer using sterile forceps.
8. Wash three times in HBSS buffer, remove excess buffer, then transfer to a 1.5 mL low-adhesion centrifuge tube.
9. Tare and record tissue weight.

***Note:*** Proceed only if: post-excision time <72 h, storage temperature maintained at 2°C–8°C, tissue mass ≥100 mg.

10. Add 200 μL chilled SCelLiVe dissociation solution to weighed tissue. Mince thoroughly with sterile scissors until paste-like consistency.
11. Add 1 mL of SCelLiVe dissociation solution to the 1.5 mL tube.
12. Use a Pasteur pipette to aspirate and expel the homogenate several times to achieve a homogeneous mixture and then transfer the entire homogenate to a 15 mL low-adhesion tube.

***Note:*** For intact biopsy specimens: Add 1 mL solution directly to minced tissue in 1.5 mL tube. For fragmented samples: Centrifuge first, then digest in 1.5 mL tube.

13. Adjust the total digestion volume according to the tissue weight by supplementing with additional SCelLiVe solution (a final volume of 2 mL per 100 mg of tissue). For instance, for a 200 mg tissue, a total of 4 mL is required; therefore, add another 2.8 mL.
14. Place 15 mL tube at 45–180° angle in preheated 37°C shaker (180 rpm).

**CRITICAL:** Monitor suspension every 15 min until complete digestion (tissue fragments fully dissociated, solution becomes cloudy).

15. Combine 10 μL cell suspension with an equal volume of 0.4% trypan blue and examine microscopically.

***Note:*** Valid suspensions show no clusters/aggregates or debris, while invalid suspensions with residual tissue fragments require additional 5–10 min digestion.

### Fluorescence-activated cell sorting of viable CD45^+^ immune cells

**Timing: 1 day**

This section aims to precisely isolate live immune cells from the heterogeneous cell suspension obtained after tissue dissociation.

16. Wash the cell suspension twice with PBS (centrifuge at 300 × g for 5 min at 4°C, discard the supernatant and resuspend in 5 mL PBS), then resuspend the cells at 1×10^6^ cells/mL in PBS for accurate quantification and subsequent standardized staining.
17. Pellet cells (300 × g, 5 min, 4°C), resuspend in Fc receptor blocking solution, and incubate for 10 min at 4°C.
18. Add anti-CD45 antibody (1 μg/10^6^ cell) and DAPI (1 μg/mL), incubate 20 min at 4°C in the dark.
19. Wash once with 2 mL PBS (300 × g, 5 min, 4°C) and resuspend in PBS for sorting.

***Note:*** The optimal staining concentration for the anti-CD45 antibody should be determined empirically for each experimental system by antibody titration.

20. Sort CD45^+^DAPI^-^ cells into PBS with 10% FBS using a 100 μm nozzle.

***Note:*** The gating strategy for cell sorting is illustrated in Figure S1, and post-sort viability should be >90% as verified by trypan blue exclusion. The percentage of residual red blood cells (RBCs) was calculated during the flow cytometry analysis setup. A gate based on FSC and SSC characteristics was used to identify the RBC population, and its percentage was determined relative to the total event count.

21. Filter sorted cells through a 40 μm strainer.
22. Centrifuge (300 × g, 5 min, 4°C).
23. Aspirate supernatant and resuspend the pellet in 1 mL PBS.

***Note:*** For optimal results, aim for a minimum yield of 1×10ˆ5 viable cells (CD45^+^DAPI^-^).

***Note:*** Maintain cells at 4°C throughout staining procedure and keep sorted cells on ice until downstream process.

### Red blood cell lysis and suspension quality control

**Timing: 1 day**

This critical quality control step removes contaminating red blood cells (RBCs) which can obscure the target immune cell population during sorting and analysis. It concludes with a rigorous assessment of cell concentration, viability, and purity to ensure that only samples meeting strict quality thresholds proceed to expensive and sensitive downstream applications like single-cell sequencing.

***Note:*** If red blood cells (RBC) account for more than 20%, perform red blood cell lysis.

24. Lysis.
  a. Transfer the cell suspension to a 15 mL or 50 mL low-adhesion centrifuge tube.
  b. Mix the cell suspension with RBC lysis buffer at a 1:2 ratio and gently invert the tube (2ml of RBC lysis buffer to 1ml of cell suspension).
  c. Incubate on ice for 5–8 min.
  d. centrifuge at 300 g for 5 min at 25°C (15°C–25°C).
  e. Carefully aspirate the supernatant using a Pasteur pipette. Remove as much supernatant as possible after centrifugation.
    ***Note:*** If RBCs exceed 80%, split the sample for separate lysis or adjust the ratio to 1:3.
  f. Resuspend the cell pellet in 1 mL of chilled PBS buffer.
  g. Examine under a microscope to confirm complete RBC lysis.
    ***Note:*** If no secondary lysis is needed, adjust the suspension volume to 10 mL with chilled PBS, gently invert to mix.
25. Centrifuge at 300 g for 5 min, aspirate the supernatant and count. troubleshooting 1.
  a. Resuspend the cell pellet in an appropriate volume of chilled PBS based on microscopic observation.
  b. Adjust the final suspension volume to approximately 10 mL. The desired working concentration for this protocol is 1–10 × 10ˆ6 cells/mL.
    ***Note:*** If the sample is too dilute, the minimum working volume is 1 mL, below which accurate handling and sorting efficiency are compromised. Concentrate the cells by centrifugation (300 × g, 5 min, 4°C) and resuspend in a smaller volume. If the sample is too concentrated, dilute stepwise with chilled PBS to remain within the recommended range. The maximum working volume is 15 mL, beyond which the cell concentration drops below the effective threshold for reproducible staining and sorting
  c. Take 10 μL of the suspension for fluorescent or 0.4% trypan blue staining (10 μL).
  d. Use a fluorescence cell counter or hemocytometer to determine cell concentration and viability.
  e. Proceed only if: Cell viability >85%;Total cell count >20,000; Impurities or RBCs <20%.

### Single-cell RNA sequencing

**Timing: 3 days**

This section describes scRNA-seq library preparation on the 10x Genomics platform, beginning with optimal cell capture (300–600 cells/μL) and GEM generation, followed by in-emulsion cell lysis, barcoding, and cDNA synthesis. It concludes with library construction using 10x Genomics kits and Illumina sequencing (PE150, ≥100,000 reads/cell), with strict quality controls at each step including cell viability assessment, cDNA amplification, and Bioanalyzer verification to ensure data reliability.

***Note:*** This methodology section outlines key steps adapted from the official 10x Genomics single-cell protocol.

26. Cell Capture.
  a. Adjust cell suspension to 300–600 viable cells/μL in PBS + 0.04% BSA (confirm concentration by an automatic cell counter, CountStar or equivalent).
  b. Load about 6,000 cells per channel onto the Chromium Chip.
  c. Run Chromium Controller per manufacturer’s protocol to generate Gel Beads-in-Emulsion (GEMs).
  d. GEM-RT Incubation.
    i. Use a thermal cycler capable of handling reaction volumes of at least 100 μL.
      ***Note:*** For the Bio-Rad C1000 Touch, set the volume to 125 μL. If using a different thermal cycler, select the maximum available reaction volume setting.
    ii. Run the retro-transcription with the following PCR table (Table 1).

**Table 1:** PCR cycling conditions

Lid temperature	Reaction volume	Run time
53°C	125 μL	∼55 min

Steps	Temperature	Time
1(first-strand cDNA synthesis)	53°C	45 min
2 (enzyme inactivation)	85°C	5 min
3	4°C	Hold
    iii. Either continue with cDNA amplification or store the samples at −20°C.
      **Pause point:** Store at −20°C for up to a week or at 4°C for up to 72 h.
27. cDNA Synthesis.
  a. Amplify barcoded cDNA using 12–14 PCR cycles (adjust based on cell input) using recommended PCR conditions from 10x Genomics protocol.
  b. Analyze cDNA using Agilent 4200 Bioanalyzer system.

***Note:*** Expected electropherogram results include (Figures S2 and S3):A broad peak corresponding to cDNA fragments of approximately 200–5000 bp. Absence of primer-dimer peaks (<100 bp).No genomic DNA contamination (which would appear >7000 bp).

**CRITICAL:** Do not exceed 14 cycles to prevent over-amplification.

28. Library Preparation and Sequencing.
29. Construct scRNA-seq libraries.
30. Follow the manufacturer’s protocol for cDNA fragmentation, end repair, adapter ligation, and library amplification.
31. Perform sequencing on an Illumina NovaSeq 6000 system with a sequencing depth of at least 100,000 reads per cell with pair-end 150 bp (PE150) reading strategy.

### Data preprocessing and basic analysis

**Timing: 3 days**

Single-cell data undergoes alignment, quality control (QC) filtering and doublet removal before normalization and Highly Variable Gene (HVG) selection. Harmony integration enables batch-corrected UMAP visualization and SNN clustering for population analysis.

***Note:*** The clustering and annotation results can be further explored and validated using interactive web tools such as Single Cell Analyst.^5^

32. scRNA-seq Data Processing and QC. troubleshooting 2.
  a. Process raw data using Cellranger v6.1.0 for demultiplexing, alignment, and quantification.
  b. Use GRCh38 human genome reference for read mapping with cellranger count.
  c. Perform quality control by filtering cells based on multiple criteria:
    i. Filter low-quality cells.
      **CRITICAL:** Remove cells with an unusually low number of detected genes.
      ***Note:*** A common cutoff is <200 genes. Adjust this threshold upward if cell viability is poor or sequencing depth is shallow.
    ii. Filter potential doublets or multiplets.
      **CRITICAL:** Remove cells with an abnormally high number of detected genes.
      ***Note:*** For PBMCs, we applied >6000 genes as a practical cutoff. Increase this value if sequencing depth is very high, or decrease it if expecting cell populations with larger transcriptomes.
    iii. Filter cells based on mitochondrial content.
      **CRITICAL:** Remove cells with a high fraction of mitochondrial gene counts, as this indicates cellular damage. We used >30% as a cutoff.
      ***Note:*** This threshold is sample-dependent. Use 10%–25% for healthy PBMCs, and up to 30%–40% for fragile or stressed tissues.
      ***Note:*** Since thresholds for filtering depend on factors such as cell type composition, source material, sequencing depth, and experimental setup, we recommend that users first inspect the distribution of QC metrics in their own dataset before deciding thresholds.
  d. Remove doublets using DoubletFinder with BCMVN-optimized pK selection.
  e. Proceed with filtered gene-cell matrix for downstream analysis.
    ***Note:*** The precise QC thresholds should always be empirically defined for each dataset. The values reported here (200–6000 genes, <30% mitochondrial reads) are representative of our PBMC datasets and are provided as an example.
33. Processing of Published scRNA-seq Datasets.
  a. Obtain published datasets from repositories, including gene-cell matrices, annotations, and metadata.
  b. Apply QC filtering (if raw data): remove cells with <200 or >6000 genes and >10% mitochondrial reads.
  c. Normalize counts using Seurat v4.2.1 (scale factor = 10,000) and log-transform.
  d. Center and scale normalized data.
  e. Select top 2000 highly variable genes (HVGs) based on mean-variance modeling.
  f. Perform PCA on HVGs for dimensionality reduction.

***Note:*** Public datasets were integrated to expand pan-cancer coverage and validate findings across malignancies.

34. Integration, dimension reduction and clustering.
  a. Perform integration using Harmony v0.1.1 on the top 30 PCA coordinates.
  b. Compute squared Euclidean distances between cell PCA embeddings (Z) and their centroids (Y) for soft clustering.
  c. Calculate batch-specific centroids and correction factors for each cluster.
  d. Generate Harmony embeddings by applying batch corrections to PCA coordinates, evaluating integration quality using PCA-corrected Harmony embeddings.
  e. Perform UMAP reduction (Seurat v4.2.1) on the first 30 Harmony embeddings.
  f. Construct SNN graph using Jaccard index (k=20 nearest neighbors) and cluster cells via SNN modularity optimization.

***Note:*** The top 30 PCs were selected based on elbow plots of explained variance, capturing >90% of biological variation. For SNN clustering, k=20 nearest neighbors balanced sensitivity to rare populations and robustness to noise, as validated by marker gene concordance.

### Mass cytometry

**Timing: 1–2 days**

**Timing: 2–3 days (for step 41)**

This section describes mass cytometry/cytometry by time-of-flight (CyTOF) analysis of Peripheral Blood Mononuclear Cells (PBMCs), beginning with Ficoll-based isolation, cryopreservation (90% FBS/10% DMSO), and metal-conjugated antibody staining, followed by fixation, intracellular staining, and acquisition through a Helios mass cytometer. Data analysis includes bead normalization, doublet filtering, X-shift clustering, and t-SNE visualization to identify distinct immune cell populations by marker expression patterns.

***Note:*** scRNA-seq identified candidate cell subsets, and CyTOF verified their existence in an independent cohort.

***Note:*** CyTOF experiments require customized antibody panels tailored to each study's specific needs. Common panel types include basic phenotyping (e.g., CD3, CD19), activation (e.g., CD25, CD69), and signaling panels (e.g., phospho-STATs). For studies validating scRNA-seq results, CyTOF panel design should occur after initial sequencing analysis. This ensures protein markers align with transcriptional findings. For example, if scRNA-seq identifies an IFN-γ-high T cell population, the CyTOF panel should include IFN-γ-related proteins.

35. Peripheral Blood Mononuclear Cells Isolation.
  a. Dilute whole blood with an equal volume of PBS, then layer this mixture over an equal volume of Ficoll solution (final ratios: blood : PBS : Ficoll = 1:1:1).
    ***Optional:*** If using granulocyte removal reagents, add to whole blood and incubate for 10 minutes at 25°C).
  b. Centrifuge for 30 min at 400 × g at 25°C with controlled acceleration/deceleration rate(set to 1–2 on a scale of 9).
  c. Carefully aspirate the buffy coat layer (white interface layer). Add 1 mL ACK lysing buffer, pipette to mix gently, and incubate for 1-2 minutes at 25°C.
  d. Add ice-cold FACS buffer (PBS with 2% FBS and 2 mM EDTA, freshly prepared) to stop lysis.
  e. Centrifuge at 400 × g for 5 min and gently resuspend in the same FACS buffer by pipetting.
  f. Resuspend cell pellet in PBS with 0.04% BSA for cell counting and viability assessment.
36. Cell Cryopreservation.
  a. Prepare freezing medium: 90% FBS + 10% DMSO.
  b. Resuspend the cells in freezing medium at a density of 5×10^6^ cells/mL by gentle mixing.
  c. Aliquot 1 mL into each cryovial. Complete the aliquoting process within 3 minutes after mixing to maintain batch consistency.
  d. Using cryo-gloves ,place the cryovials in a “programmable freezing container” and store at −80°C for at least 4 hours.
  e. Following the 4-hour holding period, place the cryovials in liquid nitrogen for long-term storage.
37. Antibody Labeling and Titration.
  a. Conjugate Antibodies to Metal Isotopes.
    i. Obtain purified antibodies from commercial suppliers (BioLegend, Thermo Fisher, Bio-Rad, R&D Systems). Antibodies must be purified, glycerol-free, and carrier-free (no BSA, hydrolyzed protein, or gelatin for stabilization).
    ii. Use the Maxpar X8 Antibody Labeling Kits to label antibodies with compatible metal isotopes to produce metal-conjugated antibodies for Fluidigm mass cytometry systems following the Maxpar Antibody Labeling User Guide.
    iii. Dilute metal-conjugated antibody to 0.2 mg/mL of stock concentration in stabilization buffer and store it at 4°C.
  b. Metal-conjugated antibodies validation and titration.
    i. Validate and titrate the conjugated antibody with relevant positive cells under specific conditions to optimize each antibody concentration.
    ii. Set up the antibody titration gradient as follows: 0(unstained), 1:50, 1:100, 1:200, 1:400.
    iii. Stain the optimized concentration of each antibody in the experimental samples.

***Note:*** Dilute metal-conjugated antibody to 0.2 mg/mL of stock concentration in stabilization buffer and store it at 4°C. For long-term storage, aliquot antibodies in small working volumes (5–10 μL) to avoid repeated freeze-thaw cycles and maintain stability.

38. Cell Thawing.
  a. Retrieve 1 or 2 cryovials and immediately thaw it/them for 2–3 minutes in a 37°C water bath. Avoid fully submerging the tube. Remove the vial when only tiny ice crystals remain.
  b. After complete thawing, move the cell suspension to a 15 mL conical tube in a biosafety cabinet.
  c. Rinse the cryovial with 1 mL of complete medium (a RPMI-1640 medium with 10% FBS. Complete it before use and preheat to 37°C) and add this solution to the conical tube containing the cells. Gently swirl the tube to mix.
  d. Make up the volume to 5mL by adding additional 3 mL of medium.
  e. Centrifuge for 5 minutes at 400 × g at 25°C.
  f. Resuspend the cell pellet in 1 mL of FACS buffer, then perform cell counting and viability assessment.
39. Cell Staining.
  a. Prepare live/dead stain (a maximum of 3×10^6^ cells per sample): wash once with 1xPBS and subsequently stain with 100 μL of Cisplatin solution for 5 min on ice to exclude dead cells.
  b. Add 1 mL FACS buffer, centrifuge at 400 × g for 5 min at 4°C. Repeat wash twice.
  c. Resuspend cells in Fc receptor blocking solution (50 μL per sample, prepared in FACS buffer) and incubate on ice for 30 min.
  d. Prepare surface antibody mix in FACS buffer (50 μL per sample). then add directly to the cells in the blocking mix (final volume = 100 μL).
  e. Resuspend and stain on ice for 30 min.
  f. Add 1 mL FACS buffer, centrifuge at 400 × g for 5 min at 4°C. Repeat wash twice.
  g. Prepare fixation buffer by mixing Intercalator solution (containing 191/193Ir, as specified in Materials Table) with Maxpar Fix and Perm Buffer (200 μL per sample).
  h. Resuspend and fix at 4°C for 8–16 hours.
  i. Add 1 mL Maxpar 1X Perm buffer, centrifuge at 800 × g for 5 min at 4°C. Repeat wash twice.
  j. Prepare pre-fixation mix using Foxp3 Fixation/Permeabilization Buffer (from the Foxp3/Transcription Factor Staining Buffer Set) (100 μL per sample). Resuspend and pre-fix at RT for 30 min.
  k. Add 1 mL Permeabilization buffer directly, centrifuge at 800 × g for 5 min at 4°C. Repeat wash twice.
  l. Prepare intracellular antibody mix in Permeabilization buffer (100 μL per sample). Resuspend and stain on ice for 30 min.
  m. Resuspend in 1 mL FACS buffer, spin at 800 × g (5 min, 4°C), and aspirate supernatant.
  n. Resuspend cells in 2 mL ddH_2_O, filter through tubes with 35 μm cell strainer cap, then centrifuge at 800 × g for 5 min at 4°C. Carefully discard supernatant.
  o. Resuspend cells in 1–2 mL ddH_2_O, take 10 μL for cell counting.
40. CyTOF Acquisition.
  a. Mix cells with 20% EQ beads for acquisition.
  b. Acquire samples on a Helios mass cytometer with standard instrument settings.

***Optional:*** To assess the effect of T cell activation on CyTOF results, cells can be treated prior to acquisition with 150 ng/mL PMA and 100 ng/mL ionomycin, followed by incubation at 37°C for 6 h.

41. Data Analysis.
  a. Debarcode samples using a doublet-filtering algorithm based on distinct elemental barcode combinations.^6^
  b. Normalize .fcs files from all batches using bead-based normalization.
  c. Cell Gating.
    i. Use FlowJo’s gating tools to sequentially remove: debris, dead cells and doublet.
    ii. Retain live, single immune cells for downstream analysis.
  d. Clustering.^7^
    i. Run X-shift clustering on single cells using multidimensional marker expression.
    ii. Adjust parameters (e.g., k nearest neighbors, iterations) to optimize biologically meaningful populations.
  e. Annotate cell types based on marker expression patterns in cluster-marker heatmaps.^8^
  f. Visualization.
    i. Project high-dimensional data into 2D space using t-SNE.
    ii. Assess cluster segregation, marker gradients, and inter-group heterogeneity.

### Crucial prerequisite: Cell type annotation

**Timing: 1–2 days**

This next section assumes that the single-cell data has been clustered and annotated. The procedures below are performed on already-defined cell populations. For complete details on the clustering and annotation process, refer to Li et al.^1^^,^^5^ The key steps were.

42. Clustering: Following data integration and dimensionality reduction (UMAP), cells were clustered using a shared nearest neighbor (SNN) graph-based algorithm (Seurat’s FindClusters() function).
43. Cell Type Annotation: Clusters were annotated into major lineages (e.g., CD4+ T cells, CD8+ T cells, Tregs, B cells, Myeloid cells) based on the expression of canonical marker genes.
44. Sub-clustering: The major CD4+ and CD8+ T cell clusters were isolated and re-clustered separately to identify finer-grained subsets (e.g., Naive, Effector, Memory, Exhausted, Cytotoxic, Tfh) based on more specific gene signatures (e.g., TCF7 for naive, GZMB for cytotoxic, TOX for exhaustion, CXCL13 for Tfh).
45. Annotation Validation: Cell type assignments were validated by Cross-referencing with established signatures from public databases and literature or independent validation using protein expression patterns from mass cytometry (CyTOF) data on a subset of sample.

***Note:*** The resulting Seurat object contains metadata columns Lineage (e.g., “CD4”, “CD8”) and Cell Type which are used for all subsequent analyses in this section. The code provided below starts from this pre-annotated object.

### Integrated multiomics analysis: Identifying response-associated T cell states and interactions

**Timing: 3–4 weeks**

This core computational section integrates the transcriptomic, proteomic, and clonotypic data generated from the previous wet-lab steps. The objective is to identify coherent cellular states, regulatory networks, and cell-cell communication patterns that are associated with response to immunotherapy, ultimately leading to the development of a predictive model.

46. Load single-cell data and splits into CD4/CD8 subsets and visualize cellular composition via UMAP. troubleshooting 3, troubleshooting 4.

This step isolates the central players in adaptive anti-tumor immunity—CD4+ and CD8+ T cells—from the fully annotated dataset and visualizes their distribution across the UMAP landscape. This provides a foundational overview of the T cell compartment’s composition before delving into deeper functional analysis.

# Load Seurat object containing pan-cancer T-cell data

>x <- readRDS("Pan_Cancer_T_Seurat.RDS")

# This pre-annotated object contains the 'Lineage' and 'Cell_Type' metadata generated in the prerequisite annotation step above.

# Subset CD4+ and CD8+ T cells

>cd4 <- subset(x, subset = Lineage == "CD4")

>cd8 <- subset(x, subset = Lineage == "CD8")

# Generate UMAP plots for both subsets

>pdf("UMAP_CD4_CD8_Cell_Types.pdf", width=10, height=8)

>DimPlot(cd4, group.by = "Cell_Type") # CD4 UMAP by cell type

>DimPlot(cd8, group.by = "Cell_Type") # CD8 UMAP by cell type

>dev.off()

47. Identify, filter, and select top-ranked significant cluster marker genes from result files.

***Note:*** Identification of Cluster-Defining Marker Genes. Prior to comparing conditions (e.g., pre- vs. post-treatment), it is essential to define the biological identity of each cell cluster. This step identifies genes that are significantly enriched in each cluster compared to all others, which are used as “markers” to annotate and understand the function of each cell population.

# Locate DEG analysis result files

>files <- list.files(path = "../DEGs/", pattern = "RDS", full.names = T)

# Initialize storage objects

>allave <- NULL # For average expression matrices

>degs <- NULL # Placeholder for DEG results (unused)

>for(i in 1:length(files)){

cname <- NULL

# Extract cell type from filename (CD4/CD8)

cname <- gsub(".(CD.)_DEGs.","\\1",files[i])

# Load and filter DEGs (adj.p<0.05, logFC>0.25)

> x <- NULL

> x <- readRDS(files[i])

> x <- x[which(x$p_val_adj < 0.05 & x$avg_log2FC > 0.25),]

> x <- x[order(x$p_val_adj, decreasing = F),]

> x <- x[order(x$avg_log2FC, decreasing = T),]

> x <- split(x, x$cluster)

# Keep top 10 DEGs per cluster

> x <- lapply(x, function(y){

>  y <- y[1:ifelse(nrow(y) > 10, 10, nrow(y)),]

> }

>x <- do.call(rbind.data.frame, x)

48. Prepare Average Expression Matrix for Visualization.

***Note:*** To generate a normalized average expression matrix for the top cluster marker genes across all cell type and treatment group combinations. This matrix is a prerequisite for creating summarized visualizations like heatmaps, which allow for the comparison of gene expression patterns across different biological conditions (e.g., Pre vs. Post-treatment) within specific cell types.

# Process based on cell type

>if(length(grep("CD4", cname, ignore.case = T)) > 0){

*>* cd4$ID <- paste(cd4$Cell_Type, cd4$Treatment_Group, sep = "_")

*>* Idents(cd4) <- "ID"

*>* ave <- NULL

*>* ave <- AverageExpression(cd4)$RNA

*>* allave[[i]] <- ave

*>* names(allave)[i] <- cname

*>* }else if(length(grep("CD8", cname, ignore.case = T)) > 0){

*>* cd8$ID <- paste(cd8$Cell_Type, cd8$Treatment_Group, sep = "_")

*>* Idents(cd8) <- "ID"

*>* ave <- NULL

*>* ave <- AverageExpression(cd8)$RNA

*>* allave[[i]] <- ave

*>* names(allave)[i] <- cname

> }

***Note:*** ‘allave’ is a list storing the average expression matrices for different CD4 or CD8 subsets.

49. Generate a heatmap of differentially expressed genes (DEGs) related to immunotherapy response.
  a. Data Preparation.
    # Filter expression matrix to include only DEGs
    >ave <- ave[which(row.names(ave) %in% unique(x$gene)),]
    # Double normalization (column and row scaling)
    >plotx <- scale(ave)   # Scale by columns (samples)
    >plotx <- t(scale(t(plotx))) # Scale by rows (genes)
    # Extract cell type info from column names
    >ctype <- unique(gsub("(CD4|CD8).","\\1",colnames(plotx)))
  b. Annotation Setup.
    i. Perform dual annotation (treatment time point and cell type).
    ii. Apply custom color schemes for annotations and expression gradients.
    iii. Prepare grouping variables for column splitting.
      # Create heatmap annotations
      >ca = HeatmapAnnotation(
      >  show_legend = c(T,T),
      >  Class = gsub(".(Pre|Post)","\\1",colnames(plotx)), # Treatment timepoint
      >  Cell_Type_Class = gsub("(CD4|CD8).","\\1",colnames(plotx)), # Cell type
      >  col = list(
      > Class = c(Pre = "#D75B58", Post = "#F5BFD3"), # Color mapping
      > Cell_Type_Class = c(CD4 = color_conditions$cold[i],
      >   CD8 = color_conditions$cold[i])))
      # Color scale for expression values
      >cols = rev(colorRampPalette(RColorBrewer::brewer.pal(11,"RdYlBu"))(100))
      # Column splitting factor
      >csplit <- gsub(".(Pre|Post)","\\1",colnames(plotx))
  c. Heatmap Generation.

>csplit <- NULL

>csplit <- gsub(".(Pre|Post)","\\1",colnames(plotx))

>somePDFPath = paste("DEGs_Heatmap_",cname,".pdf", sep = "")

>pdf(file=somePDFPath, width=6, height=4,pointsize=12)

>print(Heatmap(

> plotx,

> name = "Expression",

> column_split = csplit, # Split by treatment

> top_annotation = ca,  # Add annotations

> col = cols,   # Color scale

> cluster_columns = TRUE,

> column_names_gp = gpar(fontsize = 8),

> row_names_gp = gpar(fontsize = 0), # Hide gene names

> show_row_dend = TRUE,show_column_dend = TRUE,

> show_column_names = TRUE, show_row_names = TRUE ))

>dev.off()

***Note:*** The visualization features treatment-grouped columns with double dendrograms (genes + samples) in a compact design that hides gene names but shows sample IDs. All plots are output as PDFs with standardized dimensions.

**Figure 1:**
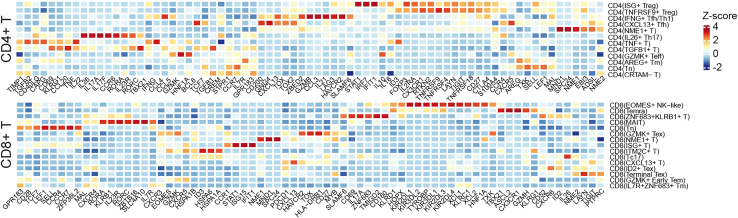
A heatmap displays z-score normalized expression levels of signature markers across identified CD4+ and CD8+ T cell subsets This figure reprinted with permission from Li et al., 2025, available under a Creative Commons CC-BY license.

***Note:*** The results of this analytical step are presented in Figure 1.

50. DotPlot of marker genes for CD4 cell and CD8 T cell subsets.
  Comparative dot plots showing signature gene expression patterns across CD4/CD8 T cell subtypes.
  a. Initialization & Data Loading.
    >cts <- c("CD4","CD8") # Cell types to process
    >pdf("Signature_Genes_CD4_CD8_DotPlot.pdf", width=10, height=14)
    >for(j in 1:length(cts)){
    # Load marker genes (convert to uppercase)
    >  cmarkers <- toupper(markers[[cts[j]]])
    # Load reference markers from file
    >  cref <- read.table(paste(cts[j],"_Markers.txt", sep="\t", header=T)
    # Select appropriate Seurat object
    >x <- if(cts[j] == "CD4") cd4 else cd8
  b. Dot Plot Configuration.

# Set cell type as identity and factor levels

>Idents(x) <- "Cell_Type"

>x$Cell_Type <- factor(x$Cell_Type, levels = sort(unique(x$Cell_Type)))

# Generate base dot plot

>p <- NULL

>p <- DotPlot(x, features = cmarkers, group.by = "Cell_Type", scale = T, cluster.idents = T) +

> theme_light(base_size = 13)+

> coord_flip()+RotatedAxis()+ggtitle(paste(cts[j], sep = ""))+

> scale_color_gradientn(colors = rev(color_conditions$RedYellowBlue))+ #ccols

> scale_size(range = c(1,6))+

> theme(panel.grid.major = element_blank(), panel.grid.minor = element_blank(), strip.background = element_blank(), panel.border = element_rect(colour = "black", fill = NA), strip.text.x = element_blank(), axis.text.y = element_text(size = 14), axis.text.x = element_text(size = 16))+

> xlab(paste(cts[j], ":Signature Markers", sep = ""))+ylab("Celltype")

# Modify percentage expression values

>p$data$pct.exp <- abs(p$data$avg.exp.scaled)

>p <- p + guides(size = guide_legend("Abs(Average Expression)"))

>print(p)

> }

>dev.off()

***Note:*** The visualization features a blue-yellow-red color gradient for expression levels with absolute value scaling, presented in a clean minimalist theme featuring proper borders and optimized font sizes for readability. All parameters including color scheme, font sizes, and scaling methods can be customized to enhance data interpretation and visual clarity.

51. Stacked barplot showing the proportional changes of cell types (‘Cell_Type’) across different cancer types, faceted by ‘IIID’ group.
  a. Data Preparation & Initialization.
    # Clear memory by removing object 'x'
    >rm(x)
    # Load pre-calculated cell type proportions
    >prop <- readRDS("Pre_Post_Prop.RDS")
    # Set up PDF output
    >pdf("Pre_Post_Barplot.pdf", width=14, height=6)
  b. Stacked Barplot Creation.

>ggplot(prop, aes(Cancer, Proportion, fill = Cell_Type, label = Cell_Type_Index)) +

> geom_bar(position = "stack", stat = "identity") + # Stacked bars

> facet_wrap(∼IIID, ncol = 4) +  # Multi-panel by patient

> scale_fill_manual(values = ccols) + # Custom color palette

> theme_linedraw(base_size = 20) + # Clean theme

> RotatedAxis() +   # Tilt x-axis labels

> theme(legend.position = "bottom", # Legend customization

> legend.title = element_blank()) +

> guides(color = guide_legend(ncol=2)) + # Multi-column legend

>  xlab("")   # Remove x-axis title

>dev.off()

**Figure 2:**
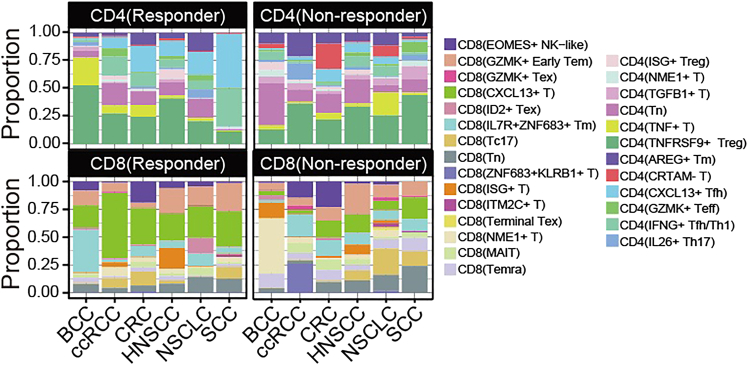
Comparative analysis of immune cell composition across six malignancies (BCC, SCC, ccRCC, CRC, HNSCC, NSCLC) stratified by ICB response status (R vs NR), presented as proportional distributions of major cell lineages This figure reprinted with permission from Li et al., 2025, available under a Creative Commons CC-BY license.

***Note:*** The results of this analytical step are presented in Figure 2.

52. Analysis and visualization of transcriptional regulatory network (Regulon) activity dynamics in CD4/CD8 T cell subsets pre- versus post-treatment, with similarity represented by phylogenetic tree clustering. troubleshooting 5.
  a. Loads pre-computed regulon activity scores (AUC) and prepares high-resolution PDF output.
    # Clear memory and load regulon AUC data
    >rm(x)
    >allaucs <- readRDS("PrePost_CD4CD8_Regulon_AUC_Celltypes.RDS")
    # Set up PDF output for tree plots
    >pdf("PrePost_CD4CD8_Regulon_AUC_Tree_Plot.pdf", width=14, height=14)
  b. Data Preprocessing.
    >for(j in 1:length(prepost)){
    > plotx <- allaucs[allaucs$Treatment_Group == prepost[j],]
    # Format sample IDs and regulon names
    > plotx$SID <- paste(plotx$Treatment_Group, plotx$Cancer, plotx$Cell_Type, sep="|")
    > plotx$Regulon <- gsub("(.) \\(.\\)$|_extended","\\1", plotx$Regulon)
    # Reshape to matrix format
    > mplotx <- reshape2::dcast(plotx, SID ∼ Regulon,
    > value.var="AUC")
    > rownames(mplotx) <- mplotx$SID
    > mplotx <- scale(as.matrix(mplotx[,-1]))
    > }
  c. Tree Construction.
    # Compute correlation-based distance
    >cdist <- cor(t(mplotx))
    >ctree <- ape::bionj(dist(cdist, method="euclidean"))
    >groupcols <- c(CD4 = color_conditions$cold[1], CD8 = color_conditions$cold[2])
    # Prepare annotation data
    >clabel <- data.frame(
    > SID = rownames(mplotx),
    > Treatment_Group = gsub("(.?)\\|(.?)\\|(.)","\\1",row.names(plotx), ignore.case = T),
    > Cancer = gsub("(.?)\\|(.?)\\|(.)","\\2",row.names(plotx), ignore.case = T),
    > Cell_Type = gsub("(.?)\\|(.?)\\|(.)","\\3",row.names(plotx), ignore.case = T))
    > clabel <- clabel[match(ctree$tip.label, clabel$SID),]
    > clabel$node <- 1:nrow(clabel)
    > cctree <- NULL
    > cctree <- full_join(ctree, clabel, by = 'node')
  d. Visualization.

# Generate tree plot

>p <- ggtree(cctree, layout="daylight", branch.length = 'none')

>clist <- NULL

>clist <- list(ctree = ctree, cctree = cctree, mplotx = mplotx, plotx = plotx, auctree = p)

>aucresults[[length(aucresults)+1]] <- clist

>names(aucresults)[length(aucresults)] <- paste("AUC", prepost[j], sep = "_")

>p <- p + geom_tippoint(aes(color=Cancer, shape = Cell_Type), size=5, alpha=0.8)+

> scale_color_manual(values = cancercols) +

> scale_shape_manual(values = 1:length(mplotx$Cell_Type))+

> geom_tiplab(aes(label = Cell_Type), size=2)+ggtitle(paste("Regulon Activity: ",prepost[j], sep = ""))+

> theme(plot.title = element_text(hjust = 0.5, size = 20))

>print(p)

> }

**Figure 3:**
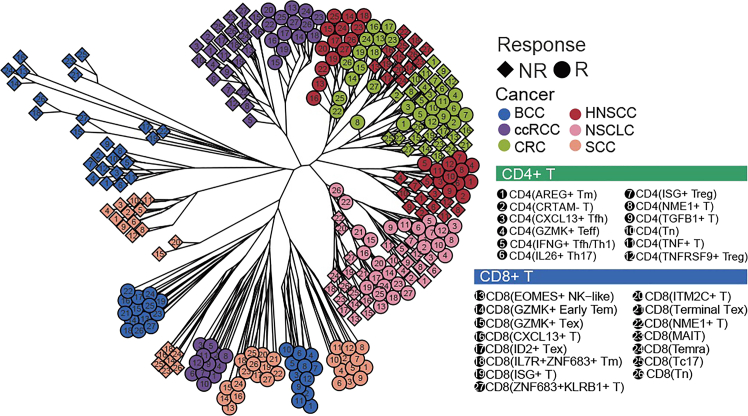
Clustering of transcription factors by their regulatory activity profiles stratifies ICB responders (R) from non-responders (NR) across multiple tumor types, revealing response specific TF modules This figure reprinted with permission from Li et al., 2025, available under a Creative Commons CC-BY license.

***Note:*** If dots in the DotPlot appear too dense, adjust the ‘size’ parameter or use faceting (‘facet_wrap’)to improve readability.

***Note:*** The results of this analytical step are presented in Figure 3.

53. Visualization of differential TCR clonal expansion.
  a. Data Loading & Preparation.
    # Load TCR clonal expansion statistics
    >tcrdata <- readRDS("Frequency_CD4CD8_Clonal_Expansion_Test_Statistics.RDS")
    >plotx <- tcrdata # Create working copy
  b. Dual Visualization.

# Plot 1: Grouped by FC categories

>p1 <- ggplot(plotx[plotx$Cutoff == 0,],

> aes(Cell_Type, Cancer, color = FC_Group, size = Sig_Level)) +

> geom_point() +

> labs(title = "Post(R) vs Post(NR)") +

> theme_classic(base_size = 20) +

> RotatedAxis()

# Plot 2: Continuous log2FC visualization

>p2 <- ggplot(plotx[plotx$Cutoff == 0,],

> aes(Cell_Type, Cancer, color = Clonal_log2FC_G2vsG1, size = Sig_Level)) +

> geom_point() +

> labs(title = "Post(R) vs Post(NR)") +

> theme_classic(base_size = 20) +

> RotatedAxis()

# Export combined plots

>pdf("Clonal_Homeostasis_Post_RNR_Specific_TBCRs.pdf",

> width=12,height=6)

>print(p1)

>print(p2)

>dev.off()

**Figure 4:**
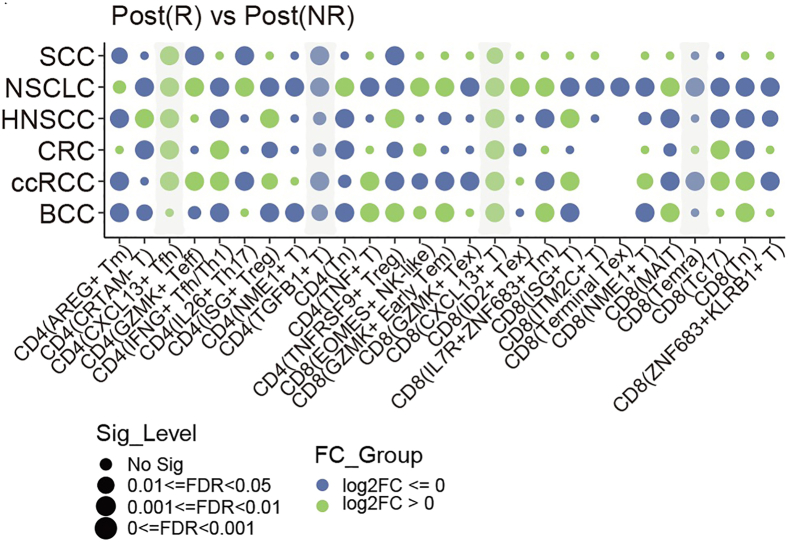
Fold changes in TCR clonotype frequencies compare responders (R; numerator) versus non-responders (NR; denominator), with Fisher’s exact test assessing significance of clonal distribution differences This figure reprinted with permission from Li et al., 2025, available under a Creative Commons CC-BY license.

***Note:*** The results of this analytical step are presented in Figure 4.

54. TCR clonal frequency: R vs NR (boxplot + stats).
  ***Note:*** The T cell receptor (TCR) repertoire diversity and clonal expansion are hallmarks of an effective immune response. This analysis tests for significant differences in the clonal frequency of expanded TCRs between patient groups (e.g., Responders vs. Non-Responders), linking specific T cell clones to treatment outcomes.
  a. Data and Parameter Setup.
    # Define comparison groups and load TCR data
    >ccompare <- list(c("Post(R)", "Post(NR)"))
    >chosen_cutoff <- 0
    >samplepostrnr <- readRDS(paste0("../TBCR/Tuning_New_Category/Cutoff_", chosen_cutoff, "_samplepostrnr.RDS"))
    # Initialize PDF output
    >pdf("FigurexxxxA2_Boxplot_Expansion0_Clonal_Homeostasis_Post_RNR_Specific_TBCRs.pdf",
    >  width=8, height=8,pointsize=12)
  b. Statistical Visualization (Individual Plots).
    # Generate per-celltype statistical plots
    >p <- NULL
    >for(i in 1:length(selected)){
    >p[[i]] <- ggbetweenstats(current[which(current$TBCR == "TCR" & current$Celltype %in% selected[i]),],
    > title = selected[i], Group, Cell_Num,
    > p.adjust.method = "BH",
    > xlab = "Post-Treatment Groups",
    > ylab = "Clonal Frequency")}
    # Combine subplots with unified title
    >combine_plots(p, plotgrid.args = list(nrow=2),
    > annotation.args = list(title= paste("TCR Clonal Expansion",sep = "")))
  c. Consolidated Boxplot Visualization.

# Create faceted comparison plot

>p <- NULL

>p <- ggplot(current[which(current$TBCR == "TCR" & current$Celltype %in% selected),],

> aes(x=Group, y=Cell_Num, color=Group)) +

> facet_wrap(∼Celltype, ncol = 2, scales = "free")+

> geom_boxplot(alpha = 0) + ggtitle(paste("TCR Clonal Expansion",sep = ""))+

> geom_point(size = 2, position = position_jitterdodge()) +

> scale_color_manual(values = ccols$general)+ ylab("Frequency")+xlab("Post-Treatment Groups")+

> stat_compare_means(comparisons = ccompare, method="t.test", label="p.format", step.increase = 0.2, hide.ns = T, hjust = -1, vjust = 1.2, tip.length = 0.1, color = "black", size = 4) + # , aes(label = paste0("p = ", ..p.format..))

> stat_compare_means(aes(group = Group), method="wilcox.test", label="p.format", hjust = 1, vjust = 0.8, tip.length = 0.1, color = "black")+

>  theme_classic(base_size = 20)+

>  theme(plot.title = element_text(face = "bold", size = 15, hjust = 0.5),

>  panel.grid.major = element_blank(),

>  panel.grid.minor = element_blank(),

>  strip.background = element_blank(),

>  panel.border = element_rect(colour = "black", fill = NA))

>print(p)

>dev.off()

55. Cell-cell interaction analysis using the ‘liana’ package, with subsequent processing and visualization of the results.
  ***Note:*** Anti-tumor immunity is a collective effort. Using ligand-receptor interaction analysis (LIANA), we predict potential communication channels between different immune cell types (e.g., T cells signaling to macrophages or dendritic cells). This helps understand how the tumor microenvironment is modulated during therapy.
  a. Loads LIANA package for ligand-receptor analysis and identifies all cell-cell interaction result files in directory.
    >library(liana)
    # Get all interaction files
    >files <- list.files(path = "CellCell/", full.names = TRUE)
  b. Data-processing.
    >for(i in 1:length(files)){
    >x <- NULL,
    >x <- readRDS(files[i])
    >x <- liana_aggregate(x) # Aggregate multiple methods
    # Standardize condition names
    >cname <- gsub(".Immune_(.)_Liana_Output.","\\1",files[i]) |>
    >   gsub("Non-responder_Post_RvsNR","Post_NR", x = _) |>
    >   gsub("Responder_Post_RvsNR","Post_R", x = _)
    # Clean cell type labels
    >x$source <- gsub("._(.)|_.$", "\\1", x$source)
    >x$target <- gsub("._(.)|_.$", "\\1", x$target)
    >x$Label <- cname
    >saveRDS(x, paste0("FigurexxxxxC_CRC_CellCell_",cname,".RDS"))
    ***Note:*** Alternatively, you can use the seq_along() function, which is sometimes safer than 1:length(x) because it won't cause errors when the length of x is zero.
  c. Significant Interaction Filtering and Visualization.

>pdf(paste0("CellCell_",cname,"_Top_20_Interactions.pdf"), width=20, height=8)

>plotx <- filter(x, aggregate_rank < 0.01) # FDR < 1%

>cts <- sort(unique(x$source)) # Get unique sender cells

>for(k in 1:length(cts)){

> if(nrow(filter(plotx, source == cts[k])) > 0){

>p <- NULL

>p <- liana_dotplot(plotx, source_groups = cts[k],

> target_groups = unique(c(plotx$source,plotx$target)),ntop=20)+

> RotatedAxis()+

*>* theme(axis.text.x=element_text(colour="black", size = 18, face = "plain"),

*>* plot.title = element_text(hjust = 0.5, size = 30))+

*>* scale_color_gradientn(colours = color_conditions$gradient)+

*>*ggtitle(paste("CRC: ",cname,sep = ""))

*>* print(p)

*>*  }}

*>*dev.off()

***Note:*** The analysis focuses on top significant interactions (FDR < 0.01), generating separate plots for each sender cell type while displaying the top 20 interactions per sender.

**Figure 5:**
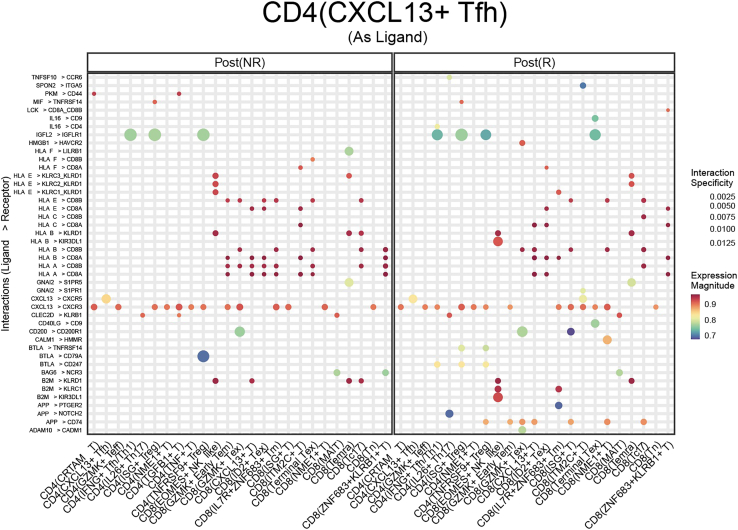
Top ligand-receptor binding interactions of CD4(CXCL13+ Tfh) Size of the dots represented interaction specificity and color gradient indicated the expression of the interaction pairs. This figure reprinted with permission from Li et al., 2025, available under a Creative Commons CC-BY license.

***Note:*** One of the results of this analytical step are presented in Figure 5.

56. Development of Response Prediction Model and Response Index (RI). troubleshooting 6.
  ***Note:*** Building a Predictive Model for Therapy Response.
  The ultimate translational goal is to integrate the multiomic features (DEGs, regulons, TCR clonality, cell-cell interactions) into a unified model that can predict which patients will respond to ICB. This step uses machine learning (elastic net regression) on the derived features to build a Response Index (RI).
  a. Perform differential expression (DE) analysis on post-ICB samples comparing responders vs. non-responders for every IRAT cell type and cancer type.
  b. Identify DEGs through a two-tailed Wilcoxon Rank-Sum test (Bonferroni-adjusted p < 0.05).
  c. Rank DEGs by log2FC and conduct GSEA (MSigDB H/C2/C6 collections) with 10,000 permutations.
  d. Select responder-enriched pathways (positive ES, p < 0.05) to construct GSEA indices per IRAT.
  e. Identify ICB-response-specific TCRs by filtering out TCRs shared with non-responders, treatment-naïve, or healthy samples.
  f. Compute clonal FC indices as log2FC of responder-specific vs. non-responder-specific TCR proportions.
  g. Split data into 70% training and 30% testing cohorts.
  h. Train GLM with elastic net (α = 0.3, 10-fold CV) using GSEA/clonal FC indices as predictors and response status (0/1) as outcome.
  i. Validate model via 100 bootstrapping rounds with random train-test splits and average AUC.
  j. Assign final response classes by majority voting across bootstrap predictions (threshold: >50% votes for “Responder”).
  k. Calculate composite GSEA index by summing TS (CD4/CXCL13+ Tfh, CD8/CXCL13+ T) and TP (CD4/TGFβ1+ T, CD8 Temra) group indices.
  l. Compute composite clonal FC index as absolute sum of TS clonal FC indices divided by sum of TP indices.
  m. Derive Response Index (RI) by multiplying composite GSEA and clonal FC indices.
    ***Note:*** Key analytical tools included Wilcoxon test, MSigDB, glmnet (α = 0.3), and caret R package, generating outputs of GSEA/clonal FC indices, GLM model, and RI thresholds.
    ***Note:*** The results of this analytical step are presented in Figure 6.
    **Figure 6:**
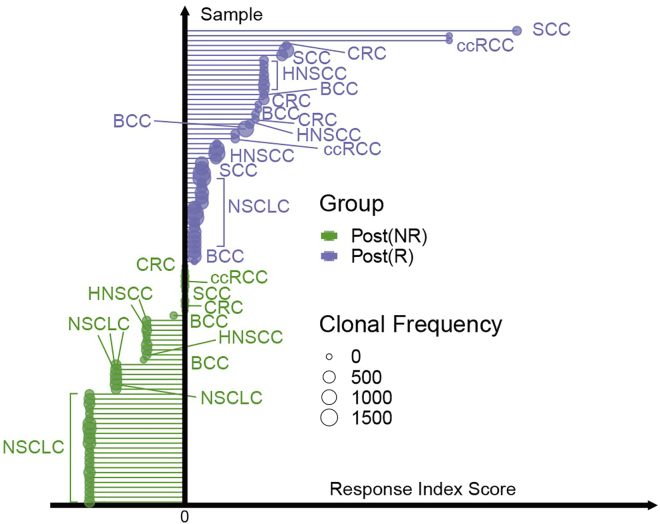
Final comparative response index across cancer types This figure reprinted with permission from Li et al., 2025, available under a Creative Commons CC-BY license.
57. Survival Analysis Based on Bulk RNA-Seq Data.
  a. Collect four post-ICB bulk RNA-Seq datasets (IMvigor210, Liu2019 SKCM, Riaz2017 SKCM, Braun2020 ccRCC).
  b. Extract expression of IRAT-specific signature markers (e.g., CXCL13, TGFβ1).
  c. Stratify patients into high or low expression groups through maximally selected rank statistics (MSRS) between 10th-90th expression quantiles.
  d. Calculate CXCL13/TGFβ1 expression ratio and stratify patients using MSRS.
  e. Group patients by TMB status (high/low) using MSRS if TMB data available.
  f. Perform Kaplan-Meier survival analysis comparing stratified groups.
  g. Assess survival differences using log-rank tests.
  h. Evaluate marker-survival associations via Cox proportional-hazards models.
  i. Generate survival curves and report hazard ratios with confidence intervals.

## Expected outcomes

This protocol enables comprehensive characterization of T cell responses to immune checkpoint blockade (ICB) through integrated multi-omics analysis. Researchers implementing this workflow can expect to generate several key datasets and analytical outcomes:

The protocol will produce high-dimensional single-cell RNA sequencing profiles from both treatment-naive and post-ICB samples, revealing therapy-associated transcriptional changes across multiple cancer types. These data enable identification of distinct T cell subsets through integrated analysis, characterizing their unique gene signatures - including activated effector populations associated with response and exhausted T cells linked to resistance.

Complementing the transcriptomic data, mass cytometry (CyTOF) analysis generates detailed protein expression profiles from PBMCs, simultaneously detecting 30–40 surface and intracellular markers. This allows validation of tumor-derived T cell subsets in circulation and discovery of potential protein biomarkers. The TCR sequencing component provides comprehensive repertoire data that tracks clonal expansion dynamics during therapy, identifies response-associated TCR sequences, and reveals significant differences in clonality between responders and non-responders.

By integrating these datasets, researchers can develop a predictive multi-omics response index (RI) that combines transcriptional signatures, TCR clonality metrics, and protein expression patterns. This RI demonstrates pan-cancer applicability for stratifying patients by likely treatment outcomes. The protocol further supports survival analysis through integration with bulk RNA-Seq data, enabling TMB-stratified outcome evaluation and validation of prognostic biomarkers.

All analytical outputs are supported by corresponding visualization tools in the provided code, with figures positioned near their generating analysis steps for easy reference.

## Limitations

This protocol has several important technical and methodological limitations that users should consider when implementation. First, the approach requires high-quality clinical specimens with strict processing timelines - optimal results depend on fresh samples processed within 1 hour of collection while maintaining >80% viability, conditions that may prove challenging in real-world clinical settings. The single-cell RNA sequencing component is inherently limited by the 10x Genomics platform’s capture efficiency (typically 50%–60% of loaded cells) and 3′-biased transcript coverage, which affects detection of full-length transcripts and certain RNA species. Similarly, the mass cytometry analysis is constrained by the current practical limit of ∼40 simultaneous metal tags and potential spectral overlap issues, restricting the breadth of protein markers that can be examined. For TCR sequencing, the protocol’s sensitivity for detecting rare clones is directly dependent on achieving sufficient sequencing depth.

A key methodological limitation lies in the multi-omics integration approach, which may produce unreliable results when: (1) applied to very small sample sizes (<5 patients per group), (2) substantial batch effects exist between different technical platforms, or (3) when samples have undergone different processing/storage conditions.

Several practical constraints should be noted: the protocol requires access to specialized equipment (Chromium controller, mass cytometer) and has substantial computational demands, particularly for the integrative analysis steps which require high-performance computing resources (minimum 32 GB RAM). The full workflow is time-intensive, typically requiring 4–6 weeks from sample processing to final analysis.

## Troubleshooting

### Problem 1

Cell counting results show high variability between replicates, or counts disagree with expected values(related to step 25).

### Potential solution

• Gently resuspend the cell suspension 10 times with a wide-bore pipette tip to prevent shear damage.
• Filter through a 40 μm cell strainer immediately prior to counting to eliminate aggregates.
• For automated counters, perform monthly calibration using standard beads.

### Problem 2

Missing Genes/Samples in Average Expression Matrix(related to step 32).

### Potential solution

Fill NA values with 0 or exclude incomplete cases.

### Problem 3

Non-Reproducibility Due to Package Versions(related to step 46).

### Potential solution

Include ‘sessionInfo()’ output at the beginning of the script and explicitly annotate critical package versions; it is recommended to use ‘renv’ for environment management or switch to Rmarkdown/Notebook to document the complete analysis workflow.

### Problem 4

Missing/Inconsistent Cluster Labels (related to step 46).

### Potential solution

Standardize labels in “meta.data” (e.g., enforce lowercase with tolower()). Use Idents() to synchronize cluster names across objects.

### Problem 5

Illogical Regulatory Network Dendrograms (related to step 52).

### Potential solution

Check if the AUC matrix contains missing values, verify whether the clustering distance metric or method settings are appropriate, and ensure consistent sample naming across all analyses.

### Problem 6

Key Ligand-Receptor Pairs Not Detected(related to step 55).

### Potential solution

Adjust the filtering thresholds or manually add known biologically significant pairs.

## Resource availability

### Lead contact

Further information and requests for resources and reagents should be directed to and will be fulfilled by the lead contact, Xuexin Li (xuexin.li@ki.se).

### Technical contact

Questions about the technical specifics of performing the protocol should be directed to and will be fulfilled by the technical contact, Xuexin Li (xuexin.li@ki.se).

### Materials availability

This study did not generate new unique reagents. All antibodies used are commercially available and listed in the key resources table. Patient-derived materials cannot be shared due to privacy and ethical considerations.

### Data and code availability

• Both the raw and processed scRNA-seq, scImmune profiling, and CyTOF have been deposited in the National Genomics Data Center under the accession number PRJCA016919.
• All other data are available in the main text or in the supplementary materials. The source code is publicly available at: https://doi.org/10.5281/zenodo.17272414.

## Acknowledgments

This research was supported by funding from the following sources: the National Natural Science Foundation of China (grants 81860499, 82473450, and 32370713), the Guangdong Basic and Applied Basic Research Foundation (grant 2021A1515220145), the International Science and Technology Cooperation Program of Liaoning Province (grant 2024JH2/101900008), the Shenyang Public Health Research and Development Technology Special (grant 81701699), the Joint Special Funds of Yunnan Province Department of Science and Technology and Kunming Medical University (grant 202401AY070001-344), and the Yunnan Major Scientific and Technological Projects (grant 202302AA310044). We sincerely thank all tissue donors for their generous contributions to this study. Special thanks also go to Uppsala University for providing computational resources and technical support through UPPMAX for this research project. The graphical abstract was created using Biorender.com.

## Author contributions

Methodology: X.L. Writing – original draft: X.J., X.Z., K.J., and W.L. Writing – review and editing: all authors.

## Declaration of interests

The authors declare no competing interests.

## Declaration of generative AI and AI-assisted technologies in the writing process

During the preparation of this work, the author(s) used DeepSeek in order to improve the readability of the language. After using this tool/service, the authors reviewed and edited the content as needed and take full responsibility for the content of the publication.
