# Supplementary material for: Protocol for decoding immune predictors of response to immunotherapy through pan-cancer multiomics analysis
Source: STAR Protoc. 2025 Nov 3;6(4):104183. doi: 10.1016/j.xpro.2025.104183 (PMC12630342; doi:10.1016/j.xpro.2025.104183)
Supplement: Document S1. Figures S1–S3 [file mmc1.pdf]

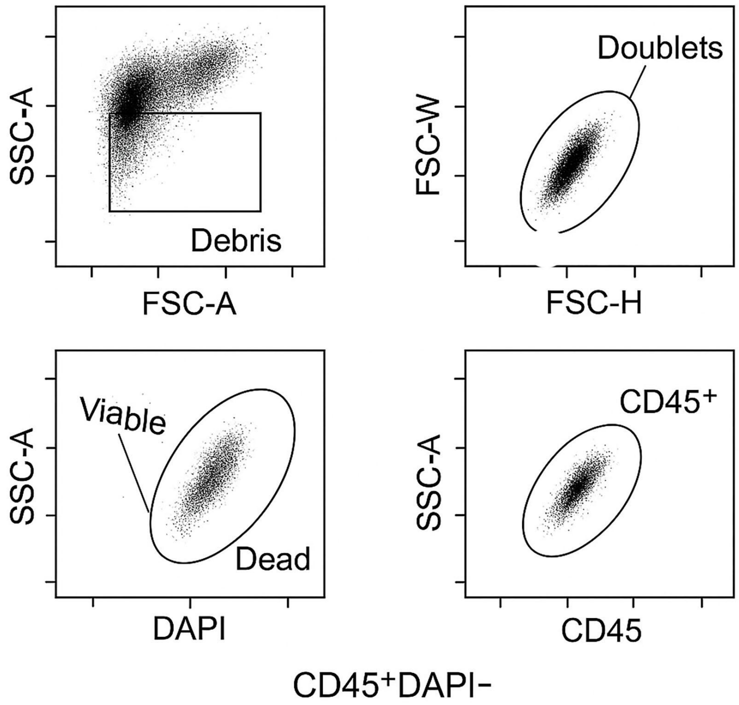

Supplementary Figure 1: Demonstration of gating strategy.Related to Step 20.

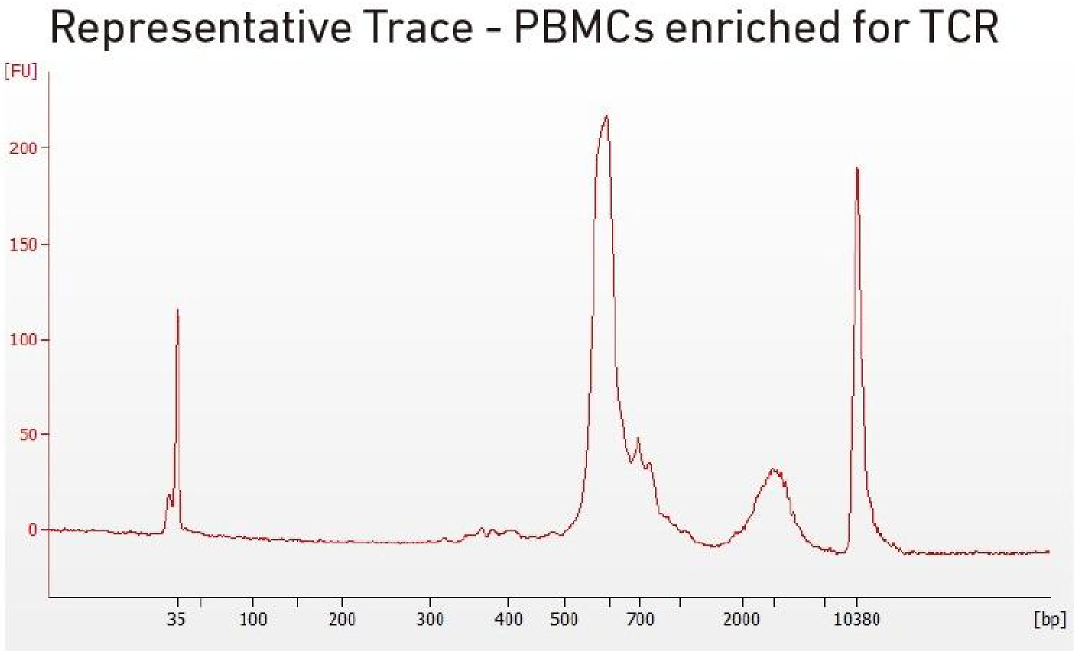

Supplementary Figure 2: Demonstration of Bioanalyzer analysis(From manufacturer).Related to Step 27.b.

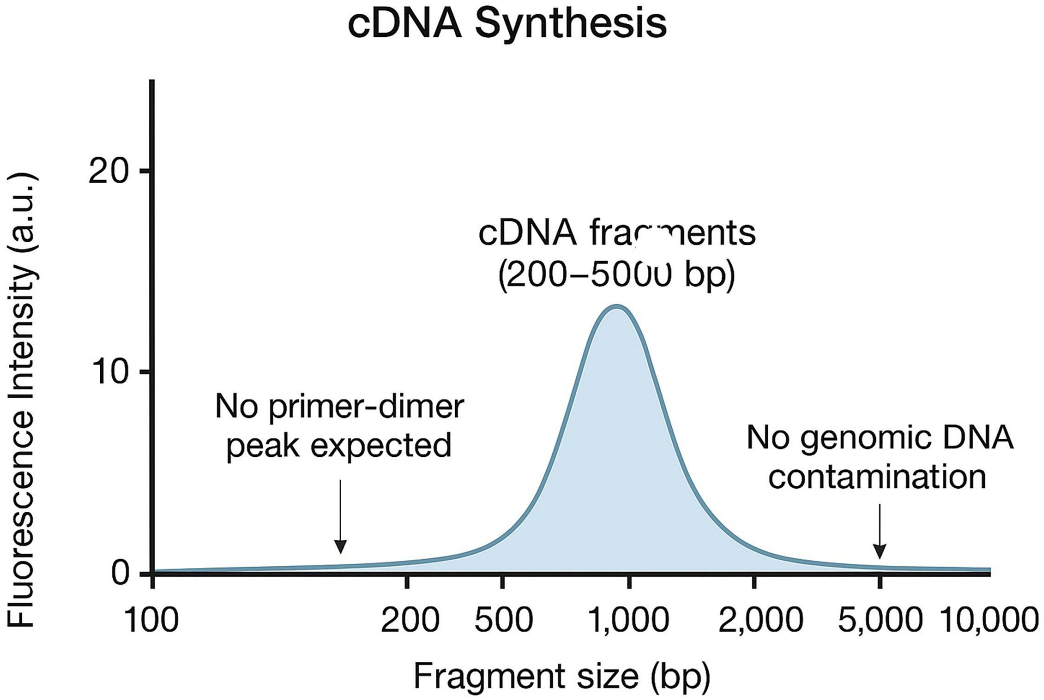

Supplementary Figure 3: Demonstration of Bioanalyzer analysis(Model).Related to Step 27.b.
